# Mechanisms of Action of Hypomethylating Agents: Endogenous Retroelements at the Epicenter

**DOI:** 10.3389/fonc.2021.650473

**Published:** 2021-03-09

**Authors:** Chryssoula Kordella, Eleftheria Lamprianidou, Ioannis Kotsianidis

**Affiliations:** Department of Hematology, University Hospital of Alexandroupolis, Democritus University of Thrace, Alexandroupolis, Greece

**Keywords:** endogenous retroelements, hypomethylating agents, 5-azacytidine, decitabine, myelodysplastic syndromes, acute myeloid leukemia

## Abstract

Abnormal DNA methylation patterns are thought to drive the pathobiology of high-risk myelodysplastic syndromes (HR-MDS) and acute myeloid leukemia (AML). Sixteen years after their initial approval, the hypomethylating agents (HMAs), 5-azacytidine (AZA) and 5-aza-2′-deoxycytidine, remain the mainstay of treatment for HR-MDS and AML. However, a connection of the hypomethylating or additional effects of HMAs with clinical responses remains yet to be shown, and the mode of action of HMAs remains obscure. Given the relatively short-lived responses and the inevitable development of resistance in HMAs, a thorough understanding of the antineoplastic mechanisms employed by HMAs holds critical importance. Recent data in cancer cell lines demonstrate that reactivation of endogenous retroelements (EREs) and induction of a cell-intrinsic antiviral response triggered by RNA neotranscripts may underlie the antitumor activity of HMAs. However, data on primary CD34^+^ cells derived from patients with HR-MDS failed to confirm a link between HMA-mediated ERE modulation and clinical response. Though difficult to reconcile the apparent discrepancy, it is possible that HMAs mediate their effects in more advanced levels of differentiation where cells become responsive to interferon, whereas, inter-individual variations in the process of RNA editing and, in particular, in the ADAR1/OAS/RNase L pathway may also confound the associations of clinical response with the induction of viral mimicry. Further *ex vivo* studies along with clinical correlations in well-annotated patient cohorts are warranted to decipher the role of ERE derepression in the antineoplastic mechanisms of HMAs.

## Introduction

In an attempt to model embryonic development, C.H. Waddington coined the term “epigenetics,” as “the causal interactions between genes and their products, which bring the phenotype into being.” (1). However, the current notion of epigenetics refers to the processes that mediate heritable changes in gene expression without changing the primary DNA sequence (2). DNA methylation, post-translational modifications of histone proteins, and post-transcriptional gene regulation by long non-coding RNA are key epigenetic mechanisms that are collectively referred to as the epigenome. The role of epigenetic alterations in promoting and maintaining cancerous growth is now well-established (3, 4). Malignant cells harbor mutations in almost all gene encoding epigenetic regulators, such as chromatin-modifying enzymes (5). In addition, the cancer epigenome exhibits aberrancies in virtually all the epigenetic control characteristics, particularly in DNA methylation, where cancer-specific hypermethylation at CpG-rich sites or CpG islands in the promoter regions theoretically lead to repression in the expression of critical tumor suppressor genes (6, 7).

Long before deciphering the complexity of DNA methylation, inhibitors of DNA methyltransferase 1 (DNMT1), the enzyme which catalyzes the addition of the methyl group to the cytosine residues, were developed (8, 9) and approved for the treatment of myelodysplastic syndromes (MDS) and acute myeloid leukemia (AML). 5-azacytidine (AZA) and decitabine (DAC) are chemical nucleoside analogs of cytidine with identical ring structure, which is attached to the ribose sugar of AZA and deoxyribose of DAC (10). Both agents induce hypomethylation after incorporation into DNA and/or RNA of highly proliferating cells and depletion of DNMT1 (11). Nevertheless, despite the wide use of the two hypomethylating agents (HMAs), the exact mechanism of action and the genetic and cellular level where HMAs exert their effects remain largely unidentified (12).

### HMAs in Myeloid Malignancies

Myelodysplastic syndromes and AML comprise two heterogeneous groups of clonal hematopoietic disorders sharing several common molecular defects (13, 14). In early 1970, AZA was first administered to patients with AML using a higher than the current dosing, which resulted in limited efficacy and severe cytotoxicity (8). Several decades later, AZA was approved for the treatment of MDS, on the basis of a less intensive regimen and of the administration *via* a subcutaneous route (15, 16). Two formulations of AZA are also approved for AML, parenteral formulation as an induction therapy for unfit patients (17), and oral formulation as maintenance treatment for patients who achieved complete remission and are ineligible for hematopoietic cell transplantation (18). Treatment with DAC was initially investigated in pediatric acute lymphoblastic leukemia (ALL) and then in MDS and AML, showing promising antitumor effects but with dose-limiting toxicities (19). Currently, DAC is approved for AML in Europe and for AML and high risk myelodysplastic syndromes (HR-MDS) in the USA (20, 21). The effectiveness, ease of use, and the favorable toxicity profile have rendered HMAs as the backbone for combination regimens for clinical trials in AML and MDS. However, the median overall survival with HMA monotherapy is ~13–16 months for patients with MDS (22) and less than a year for the ones with AML (17). In addition, both primary and secondary, i.e., after an initial response, failure to HMAs confer a grave (23–25) outcome, and there is currently no approach to overcome the inevitable development of resistance to HMAs (26).

### Immune Mechanisms of Action of HMAs

Hypomethylating agents induce global hypomethylation and purportedly have pleiotropic effects. Beyond the presumed derepression of tumor suppressor genes, HMA-mediated hypomethylation potentially affects cell cycle control, DNA repair, apoptosis, cell signaling, angiogenesis and control of cancer cell invasion, and metastasis (27, 28). In addition, the dissociation between the degree of HMA-induced demethylation and clinical response (29) points to alternative, DNMT-independent mechanisms, such as direct cytotoxicity *via* inhibition of protein synthesis and activation of DNA damage pathways (30) and immunomodulation. Epigenetic silencing of immune-response genes by DNA methylation characterizes the cancer genome (31), and HMAs can restore numerous pathways of cancer immune evasion (32). Consequently, the antileukemic activity of HMA could be, at least partially, immune-mediated, but literature reports are often contradictory. HMA can promote the antitumor response by several mechanisms encompassing increased tumor immunogenicity and enhanced the cellular- and cytokine-mediated effector T-cell tumor lysis (32, 33). Conversely, HMA may inhibit T-cell proliferation and pro-inflammatory cytokine secretion with simultaneous induction of regulatory T cells (Tregs) (34, 35), while the HMA-induced increase in the expression of immune checkpoint molecules in clonal CD34^+^ progenitors is associated with refractory disease in patients with high-risk MDS (36). Partially reconciling these antithetic effects, another study demonstrated that HMAs are probably effective only in tumors with an “immune evasion” gene expression signature (37). HMAs can also directly affect leukemic and immune signaling pathways, and perturbed signaling networks in malignancies are not only detected in cancer cells but also in the cellular components of tumor immunity (38, 39). We have shown that the AZA-mediated restoration of the pathological signal transducer and activator of transcription (STAT) biosignature in both CD34^+^ and CD4^+^ T cells is strongly linked with a favorable clinical outcome in patients with high-risk MDS (40, 41). However, in accordance with the selective immunological activity of HMAs, no effect on the STAT networks was observed in patients who were refractory to AZA. Though not readily interpretable, the highly diverse clinical course of patients treated with HMA (42) and the equally heterogeneous, sample-specific responses to HMAs observed in multiomics studies (43, 44) argue for a multifaceted mechanism of the action of HMAs, potentially influenced by the molecular background of individual patients.

Another candidate antineoplastic mechanism of HMAs is the reactivation of human endogenous retroelements (EREs) (45) and the induction of viral mimicry (46). Recently, several intriguing reports rekindled interest on the ERE-induced antitumor immune response by demonstrating the upregulation of endogenous double-stranded RNAs (dsRNAs) and the induction of type I and III interferon (IFN) responses- in cancer cells treated with epigenetic agents (47–53).

### Endogenous Retroelements

Endogenous retroelements comprise a significant part of the human genome, composing about 43% of the genome (54). EREs are distinguished into non-long terminal repeats (non-LTRs), which include the long and short interspersed nuclear elements (LINEs and SINEs), and LTR elements. With the exception of about 100 young elements, the rest of the LINEs reside as inactive fragments (55). The most common SINEs and the more abundant mobile elements in the human genome are Alu elements which comprise up to 11% of the genome. Alus have a variable impact on gene functions, and up to 0.3% of all human genetic disorders are associated with Alu-mediated recombination (56). Human endogenous retroviruses (HERVs) and mammalian apparent LTR retrotransposons (MaLRs) are part of the LTR elements (57–59). HERVs are remnants of germ-line integrations of exogenous retroviruses which in the past have infected the host (57, 60) and occupied ~8% of our genomes (54). ERVs are the only EREs that could be transmitted from one cell to another (60, 61). However, most of the HERVs have lost this ability due to mutations and recombination events that have been accumulated for years during the evolution. Recently acquired HERVs have preserved their copies intact and retain the ability to produce infectious particles, while evolutionarily old LTRs are more likely inactivated and not replication-competent (62).

### EREs and Leukemogenesis

Since the first discovery of transposable elements (63), hypothetical links between repetitive elements and tumorigenesis have started to emerge, and a multitude of ERE-mediated mechanisms ultimately leading to the disruption of genomic integrity have been reported (57, 64, 65). The term “onco-exaptation” has been coined to describe the exploitation of the epigenetic and transcriptional dysregulation in cancer by EREs (66). Reactivation of EREs may promote carcinogenesis *via* the activation of cryptic promoters and the formation of chimeric transcripts with gene regulatory properties (64, 67). Specifically, the data for leukemogenesis are scarce, and a direct demonstration of leukemogenic potential of EREs in the primary AML cells is still lacking. Rearrangements of the MLL gene in AML have been associated with Alu-mediated recombination events (68–70), while the induction of AML in a xenograft mouse model for primary myelofibrosis (PMF) was attributed to unrestricted replication and subsequent viremia of murine leukemia virus (MuLV), potentially due to a paracrine mechanism in peptide mass fingerprinting (PMF) (71). A recent study demonstrated that specific HERVs can also act as oncogenic enhancers in AML. Using genome and epigenome editing approaches in AML cell lines, the authors detected six ERV families which bore chromatin signatures of enhancers and could regulate the host gene expression. It is to be noted that the deletion of the AML-specific LTR2B elements decreased proliferation and induced the apoptosis of AML cell lines by reducing the expression of the apolipoprotein C1 (*APOC1*), an oncogene (72).

### EREs, Tumor Immunity, and HMAs

In contrast to their putative role in oncogenesis and leukemogenesis, EREs can potentially promote antitumor immunity. Epigenetic deregulation in tumors results in the expression of several ERE antigens not expressed in healthy tissues which can, at least theoretically, trigger immune sensing and induce potent adaptive antitumor responses (58). Cytotoxic CD8+ T-cell responses against epitopes of certain HERV proteins have been reported in several tumors (73–76), and the *in silico* analysis of local cytolytic activity in 18 untreated tumors identified a set of three tumor-specific ERVs (TSERVs), untraceable in the corresponding normal tissues (77). Importantly, the immune pathways were enriched in tumors with the highest expression of TSERVs, whereas, the cytolytic activity in several tumor types correlated with the expression of other HERVs. Upregulation of EREs in AML was linked to specific mutations, but, rather unexpectedly, not with *DNMT3A* and *TET2* mutations (51, 78). *IDH1* and *TP53* mutations were associated with suppression in the expression of ERE (78), consistent with the low immunogenicity of *IDH1*-mutated tumors (79) and the role of *TP53* dysfunction in tumor-immune evasion (80). Activating mutations of the SET-binding protein 1 gene (*SETBP1*) and the overexpression of wild-type *SETDB1* are associated with aggressive diseases and poor outcomes in myeloid neoplasms (81). The disruption of *SETDB1* in AML cell lines triggers a type-I IFN antiviral response by desilencing both the LTR and non-LTR elements, indicating that the evasion of innate immune sensing of EREs possibly underlies the poor prognostic impact of *SETDB1* alterations in AML and MDS (51).

Regarding the therapeutic derepression of EREs, Jaenisch et al. (45) first showed that AZA can reactivate silent retroviral genomes in mice, whereas, in 1999, Karpf et al. (82) reported the induction of IFN responsive genes by AZA in HT29 colon adenocarcinoma cells. Two recent articles revisited these phenomena and addressed their role in the antineoplastic mechanisms employed by synthetic azanucleosides. Utilizing the *in vitro* assays in colorectal cancer cells (CRCs), Roulois et al. (47) demonstrated that a low dose of DAC can induce the formation of dsRNAs into cancer-initiating cells (CICs). These dsRNAs were mainly derived from endogenous retroviral elements and activated the melanoma differentiation-associated protein 5 (MDA5), a cytosolic pattern recognition receptor, the mitochondrial antiviral-signaling protein (MAVS), its downstream signaling modules, and IFN regulatory factor 7 (IRF7). Triggering the MDA5/MAVS/IRF7 axis culminates in the induction of an IFN type-III response, upregulation of IFN-stimulated genes (ISGs), and setting the CICs into a “virus-infected” state. Analogous findings were reported by Chiapinelli et al. (48), who observed a HMA-mediated induction of IFN type-I response in ovarian cancer cell lines *via* ERV demethylation and dsRNA formation. Also, AZA-induced viral defense gene levels discriminated epithelial ovarian cancer tumors into good (high levels) and poor (low levels) prognosis, whereas, an intense viral defense signature was associated with a better outcome in patients with melanoma treated with the anti-CTLA-4 therapy. Given the pleiotropic function of IFNs in immune response (83), the authors further used murine models to show a synergistic effect of the combination of AZA with anti-CTLA-4 antibody (48).

In line with the above reports, the antitumor activity of other agents targeting the epigenetic machinery was also based on the induction of ERE-mediated viral mimicry. Experiments in breast cancer cell lines and patient-derived xenograft models revealed that CDK4/6 inhibitors upregulate the *ERV3-1* gene by reducing the activity of DNMT1, leading to a type-III IFN viral mimicry response (49). Ablation of the histone demethylase LSD1 in cancer cell lines also induced the reexpression of HERVs, LINE1, and the AluYa5 sub-family of Alu elements, which in turn mediated a dsRNA-diven type-I IFN antitumor response, without affecting either global methylation or the DNMT1 levels (50). Moreover, the derepression of LINE1 by histone deacetylase (HDAC) inhibitors led to the increased death of drug-resistant cancer cell lines by permitting a chemotherapy-induced antiviral defense response (52), whereas, the combination of epigenetic therapies enhanced the viral defense response and increased the antitumor efficacy (84, 85). However, the global patterns of the reexpression of ERE and the heterogeneous investigational approaches of the above studies hampered the identification of the exact origin of drug-induced immunogenic EREs. By using an MDA5-protection assay and RNA-sequencing (RNA-seq) in CRCs, Mehdipour et al. (53) identified inverted-repeat Alus (IRAlus) as the major source of DAC-induced immunogenic dsRNAs. Almost 90% of the MDA-protected RNA was IRAlus, whereas, the LTR elements were represented only by 1.37% and the ERV-derived dsRNAs had no role in the induction of viral mimicry (53).

In contrast to the majority of the abovementioned studies that used cancer cells lines or murine models, we investigated the effect of AZA on the pattern of ERE expression on CD34^+^ primary hematopoietic stem cells (HSCs) derived from patients with MDS undergoing treatment with AZA (86). By using RNA-seq, sophisticated bioinformatics tools, and the *de novo* assembly, we charted a complete transcriptional profile of EREs in the bone marrow of HSCs from healthy donors and patients with AML, MDS, and chronic myelomonocytic leukemia (CMML), before and after AZA administration. Even though the transcription of EREs increased after six cycles of AZA, this effect was equally observed in both patients with complete remission (CR) and failure to AZA. An analysis of an independent dataset (accession number: SRP067631) in a comparable cohort (87) revealed identical results, clearly suggesting that the response to AZA cannot be predicted by the global upregulation of EREs. We further analyzed the AZA-mediated modulation of specific ERE groups of loci, but, again, we were unable to track significant differences based on the treatment response. To address the possibility of an ERE-induced antiviral response only in patients with CR, we also assessed the alterations of IFN-inducible LTR elements or IFN-signature genes (ISGs) in our patients and the aforementioned cohort (87). No induction after AZA therapy or correlation with its outcome was observed either after six cycles of AZA or as early as day 15 after the first cycle of AZA, thus pre-cluding a sustained IFN signature in HSCs as a mechanism of the AZA activity. In keeping with our results, no correlation between an IFN response in the bone marrow of CD34^+^ cells and the response to AZA and DAC was shown in a cohort of 55 patients with MDS and CMML, whereas, the inflammatory signaling was reduced instead of enhanced after HMA administration (88). Also, in a heterogeneous cohort of patients with myeloid and lymphoid malignancies, evolutionarily young EREs were pre-ferentially upregulated in responders to AZA. However, the same EREs were also upregulated in few non-responders, whereas, the analyses were performed in diverse cell subpopulations, thus adding another layer of complexity to the interpretation of the findings (89). Of note both *in vitro* (48) and in patients treated with HMAs (88), no differential regulation of EREs or induction of an IFN response was noted between AZA and DAC, whereas, guadecitabine also appears to induce an ERE-mediated immune response, but this has not been formally shown (90).

**Figure 1:**
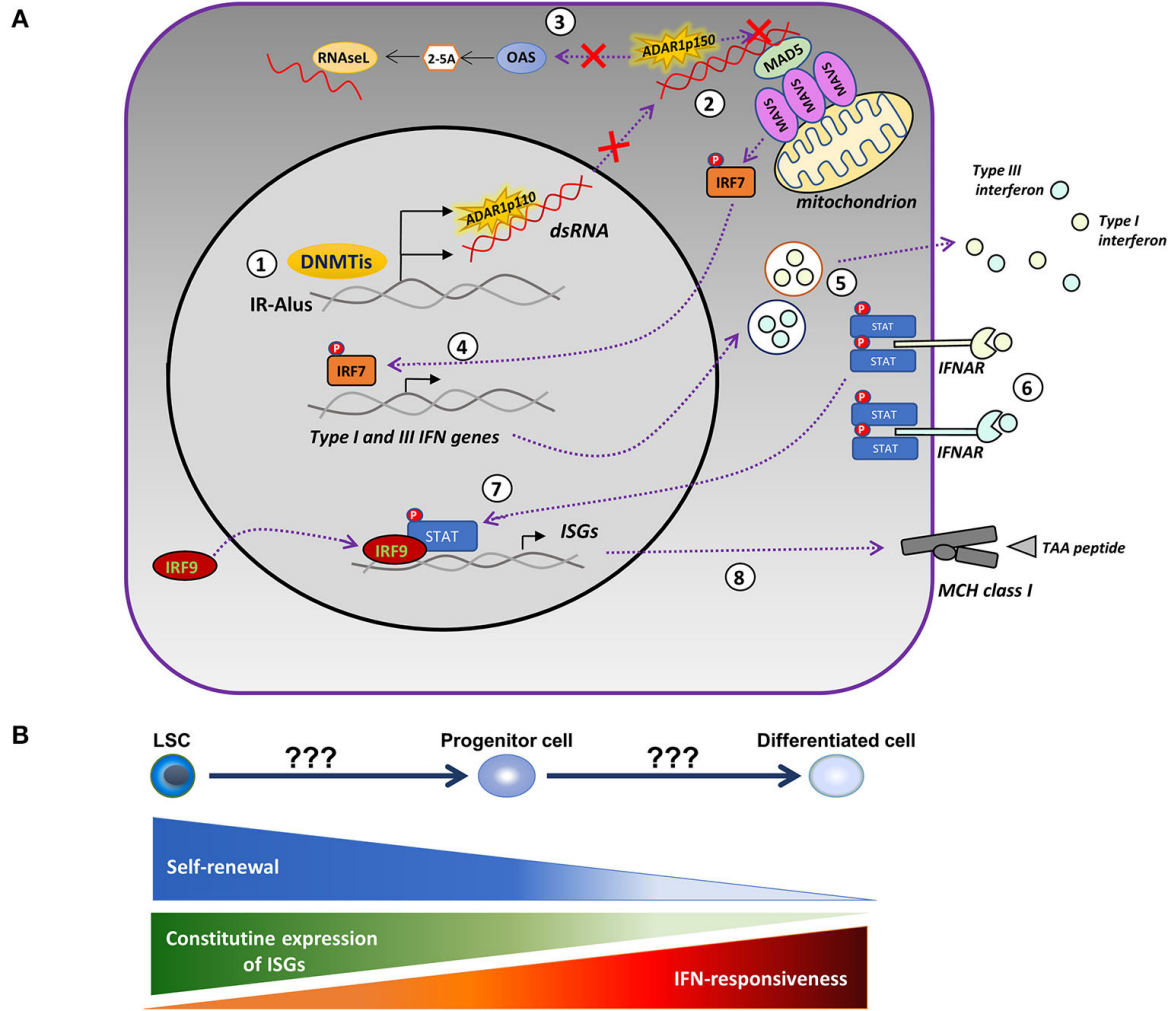
Modified model of the HMA-induced viral mimicry in leukemic cells. **(A)** Treatment with DNA-methyltransferase inhibitors (DNMTis), such as azacytidine (AZA) and decitabine (DAC), reactivate repressed inverted-repeat Alus (IRAlus), leading to the formation of double-stranded RNAs (dsRNAs). The same agents upregulate the expression of the RNA-editing enzyme, ADAR1 (1). The pattern recognition receptor, MDA5, senses the dsRNAs located in the cytoplasm, leading to the aggregation of the mitochondrial antiviral-signaling protein (MAVS) and to the phosphorylation of interferon (IFN) regulatory factor 7 (IRF7). However, increased ADAR1 activity depletes dsRNAs and prevents the activation of MDA5 (2), whereas, it further inhibits the OAS/RNAse L apoptotic pathway (3). In the absence of ADAR1 upregulation, activated IRF7 translocates into the nucleus inducing the transcription of type-I and III IFN (4), which are then secreted into the tumor microenvironment (5) and bind to their receptors, causing the phosphorylation of signal transducer and activator of transcription (STAT) proteins (6). Activated STATs associate with the IRF9 and move into the nucleus (7), where they induce the expression of IFN-stimulated genes (ISGs) and the major histocompatibility complex (MHC) molecules (8) by increasing the ability of tumor cells to present tumor-associated antigens (TAAs). **(B)** The competence of HMAs to induce a viral mimicry state potentially depends on the level of cellular differentiation. The latter defines the permissiveness to IFN-mediated induction of ISGs and the degree of upregulation of ADAR1.

The discrepancy of findings in the pre-clinical data of patients with MDS might be in part due to the intrinsically high constitutive expression of ISGs and the resistance of HSCs to IFN stimulation (91). In contrast, the differentiated cells downregulated the expression of ISGs and became responsive to IFN (91), indicating that the level of cellular differentiation might be an important factor in determining the mode of action of HMAs. Another possibility is the antagonizing role of the ADAR1 enzyme in the cytotoxic effect of AZA. ADAR1 edits dsRNA through the conversion of adenosine to inosine, resulting in the destabilization of RNA duplexes (92), repression of the 2′,5′-oligoadenylate synthetase (OAS)-RNase L pathway (93), and blocking the activation of the MDA5 receptor (94). Inactivating mutations of ADAR1 are present in Aicardi–Goutières syndrome (AGS), an autoimmune disorder, and are accompanied by an intense type-I IFN signature and upregulation of ISGs (95). Knockout of the IFN-inducible ADAR1 p150 isoform in a lung adenocarcinoma cell line enhanced the cytotoxic potential of AZA *via* reactivation of the single-stranded RNA-specific endoribonuclease RNAase L, an antiviral enzyme (93). Mehdipour and colleagues (53) also elegantly showed that the efficacy of HMAs is interdependent on the expression of ADAR1 and activity. DAC induced the expression of ADAR1 in CRCs and depleted immunogenic dsRNAs, while ADAR1-knockdown of both the constitutively expressed (p110) and the interferon-inducible (p150) forms resulted in persistent upregulation of ISGs and an enhanced activation of MAVS. Further confirming the bidirectional interaction between HMAs and ADAR1, a low dose DAC in CRC xenograft models was efficacious only in mice injected with ADAR1-knockdown CRCs. Collectively, the above data suggest that the differential expression of ADAR1 enzyme and/or regulators of the ADAR1/OAS/RNase L pathway may account for the disconnection between the clinical response and the induction of viral mimicry observed in our study. Several tumors express high levels of ADAR1 (96) and display dysregulated RNA editing (97), but, in others, the depletion of the ADAR1 may instead promote tumor progression and metastasis (98, 99). In AML, ADAR1 is variably expressed (100) and a JAK2-dependent upregulation of ADAR1 p150 takes place during blast crisis in chronic myeloid leukemia (CML) (101), but there is a lack of correlations between ADAR1 levels and activity and clinical outcomes. Importantly, the expression and activity of ADAR1 increases during the differentiation of myeloid cell lines (100); therefore, measurements on more differentiated myeloid forms rather than the leukemic blasts will probably be more relevant for clinical associations. Based on the above considerations, an updated model of the viral mimicry hypothesis is provided in Figure 1.

## Conclusion

More than 50 years after their discovery, HMAs remain as the only approved compound for the treatment of HR-MDS and the main therapeutic option for unfit patients with AML. Paradoxically, after 16 years of clinical experience, the mechanism of action of HMAs is still under investigation, a fact that poses obvious obstacles in bypassing the resistance and developing rational combinations with other agents. Endogenous retroelements, once viewed as parasitic elements, are currently enjoying a resurgence of interest regarding their role in the mechanism of action of HMAs. Aside from its potential use as a predictor of response to immunotherapy, the HMA-mediated induction of immunogenic EREs appears to sensitize immune-refractory tumors to checkpoint inhibition. However, a robust clinical proof confirming a cause–effect relationship of the induction of viral mimicry with the efficacy of HMAs is currently lacking. In addition, the combination of HMAs with immune checkpoint inhibitors demonstrates the modest efficacy in clinical trials for patients with MDS (102). While a multitude of issues pertaining to treatment schedule, dosing, and pharmacological attributes may account for the discordance between research findings and clinical efficacy, the interpatient-diversity of the tumor-immune system interactions is an obvious obstacle that has to be thoroughly interrogated before assigning a mechanistic role of ERE reactivation in the clinical activity of HMAs. A deeper understanding of the regulation of HMA-mediated reactivation of EREs at the single cell level and large-scale correlations of the experimental findings with clinical information is required to circumvent the limitations of both HMA and immune therapy in myeloid neoplasms.

## Author Contributions

CK and IK wrote the manuscript and created the figure. CK, EL, and IK revised the manuscript. All authors contributed to the article and approved the submitted version.

## Conflict of Interest

IK have received research funding from Celgene Corporation and received honoraria from Genesis Pharma Hellas. The remaining authors declare that the research was conducted in the absence of any commercial or financial relationships that could be construed as a potential conflict of interest.
